# Concordance of adenosine deaminase with immunoglobulins and lymphocyte subsets in EBV-related diseases

**DOI:** 10.1186/s13052-023-01457-0

**Published:** 2023-04-24

**Authors:** Ting Shi, Qi Ding, Xinglou Liu, Guo Ai, Hua Zhou, Linlin Huang

**Affiliations:** 1 Department of Infectious Diseases, Children’s Hospital of Soochow University, 303 Jingde Road, Suzhou, 215000 Jiangsu China; 2 Department of Dermatology, Medical School, Affiliated Hospital of Nantong University, Nantong University, Nantong, Jiangsu China; 3 Department of Pediatrics, Tongji hospital, Tongji Medical College, Huazhong University of Science and Technology, Wuhan, Hubei China; 4 Pediatric Intensive Care Unit, Children’s Hospital of Soochow University, Suzhou, Jiangsu China

**Keywords:** Adenosine deaminase, Epstein-Barr virus, Immunoglobulin, Lymphocyte subset

## Abstract

**Background:**

Clinical manifestations of Epstein–Barr virus (EBV) infection are diverse. This study aimed to explore the immune response in EBV-related diseases and the correlation between immune cells and adenosine deaminase (ADA) levels.

**Methods:**

This study was conducted at the Children’s Hospital of Soochow University. In total, 104 patients with EBV-associated respiratory tract infection (EBV-RTI), 32 patients with atypical EBV infection, 54 patients with EBV-associated infectious mononucleosis (IM1, with normal alanine aminotransferase [ALT] levels), 50 patients with EBV-IM2 (with elevated ALT levels), 50 patients with acute respiratory infection (AURI, with other pathogens), and 30 healthy controls were enrolled in this study. Indicators of ADA, immunoglobulins (Igs), and lymphocyte subsets were analyzed for EBV-related diseases.

**Results:**

Differences in the white blood cell, lymphocyte counts, ADA levels, IgA, IgG and IgM titers, percentage of CD3^+^, CD3^+^CD4^+^, CD3^+^CD8^+^, CD16^+^CD56^+^, CD3^−^CD19^+^, and CD19^+^CD23^+^ lymphocytes, and CD4^+^/CD8^+^ ratio between EBV-related disease groups were all statistically significant (*P* < 0.01). ADA levels in the EBV-related disease groups were significantly higher than those in the control group (*P* < 0.01). The lymphocyte count, ADA levels, IgA and IgG titers, and percentage of CD3^+^ and CD3^+^CD8 + lymphocytes in the atypical EBV infection, EBV-IM1, and EBV-IM2 groups were significantly higher than those in the EBV-RTI, AUTI, and control groups (*P* < 0.01), whereas the percentage of CD3^+^CD4^+^, CD3^−^CD19^+^, and CD19^+^CD23^+^ lymphocytes and CD4^+^/CD8^+^ ratio showed the opposite trend. ADA levels were consistent with and closely related to the viral load and cellular and humoral immunity in EBV-related diseases.

**Conclusions:**

ADA levels, humoral immunity, and cellular immunity were diverse in EBV-related diseases, and ADA was closely related to Igs and lymphocyte subsets.

## Background

Epstein–Barr virus (EBV) is a gamma herpes virus that is highly prevalent worldwide. More than 90% of the world’s adult population is infected with the EBV [1]. EBV can infect epithelial cells, enter circulating B lymphocytes, and persist in a latent state [2]. Most infections in young children were benign and subclinical. However, 50% of EBV infections manifest as infectious mononucleosis (IM) during adolescence [3]. EBV has been associated with various diseases, such as respiratory infections, encephalitis, autoimmune diseases, immune dysfunction, malignant lymphoma, aplastic anemia, nasopharyngeal carcinoma, and hemophagocytic lymphohistiocytosis, in addition to IM [4]. The symptoms of EBV infection vary according to the viral load, immune status, and age of the patient [5, 6]. In our previous study [5], the most prominent disease caused by EBV infection in hospitalized children was IM, followed by respiratory tract infection (RTI) and atypical infection.

Currently, the immunopathological mechanism of acute EBV infection involves the infection of B lymphocytes by the virus and its entry into the bloodstream. The expression of EBV viral capsid antigen (VCA) was accompanied by the production of immunoglobulin M (IgM) and IgG and an extraordinary expansion of CD8^+^ T lymphocytes [7]. The CD8^+^ cytotoxic T cells played a critical role in IM virus infection of B lymphocytes [8]; however, excessive immune responses contributed to organ damage. In addition, antibody production and efficient generation of cytotoxic CD8^+^ T cells must be assisted by MHC class II-restricted CD4^+^ T cells [9]. Recently, an increasing number of studies have shown that natural killer (NK) cells preferentially recognized lytic replication and proliferate during IM [10]. However, it is unclear why some individuals present with IM upon primary EBV infection, whereas others present with acute fever or respiratory infections.

Adenosine deaminase (ADA) was a key enzyme in the adenosine metabolic pathway and widely distributed in cells of various tissues in the human body, especially abundant in lymphoid tissues. It reduced the levels of intracellular adenosine as well as 2-deoxyadenosine and protected cells from apoptosis. In addition, extracellular ADA can bind to cell surface anchored proteins and function as a costimulator, allosteric modulator, and intercellular signaling molecule[11]. Previous studies [12] have confirmed that ADA played a vital role in the differentiation and growth of lymphocytes, macrophages, and NK cells. It was considered a marker of T-lymphocyte-mediated immunity. In 1984, Mejer et al. [13] found that ADA expression was elevated in EBV-IM. Additionally, in our previous study [14], ADA demonstrated a good diagnostic value for EBV-IM in febrile patients and was an indicator of EBV-IM severity in children. This study will further explores the differences in ADA levels in EBV-associated diseases and the correlation between ADA and Igs and lymphocytes. Our findings will help clinicians understand EBV-associated diseases in children and avoid the occurrence of missed diagnoses, as well as provide clues for clarifying the immunopathological mechanisms of EBV-associated diseases.

## Methods

### Data sources and study design

This prospective study was conducted at the Children’s Hospital of Soochow University between May 2018 and December 2019. The case group included hospitalized children with cough and/or fever. Patients with mixed infections; autoimmune diseases; a history of immunosuppressive, immunomodulatory, or antiviral drug intake in the past 2 weeks; chronic diseases; and immunodeficiency diseases were excluded. The control group consisted of healthy children who underwent surgery (inguinal hernia and phimosis), were unaffected for the last 2 weeks, and had no history of taking medications. Routine blood tests and assays for detecting EBV-specific antibodies, plasma EBV-DNA, ADA, alanine aminotransferase (ALT), lymphocyte subsets, and Igs were performed within 24 h of admission. A total of 320 participants (162 males and 158 females; age range, 0.7–12.2 years) were enrolled in this study. IM patients without ALT level elevation served as the IM1 group, and children with IM accompanied by the elevation in ALT levels served as the IM2 group. The mean ALT value of children in IM2 group was 130.0 ± 114.6 U/L.This study was approved by the Ethics Committee of Children’s Hospital of Soochow University, China (No.2019KS004). All the participants provided written informed consent.

### IM and atypical EBV infection

The criteria for IM were as follows [15]: (1) meeting more than three clinical manifestations: fever, tonsillopharyngitis, cervical lymphadenopathy, splenomegaly, and hepatomegaly; (2) presence of IgM and IgG to EBV VCA (VCA-IgM- and VCA-IgG-positive, respectively), with absence of IgG to EB nuclear antigen (EBNA1), or VCA-IgM-negative and VCA-IgG-positive (low affinity); and (3) exclusion of other infections, such as human immunodeficiency virus and cytomegalovirus.

Atypical EBV infection was defined as the onset of fever and/or elevated atypical lymphocytes in the peripheral blood, without target organ damage.

### Laboratory assays

#### Routine complete blood count and determination of ADA, ALT, IgG, IgM, and IgA levels

Routine blood counts were performed using a BC-5310 instrument (Shenzhen Mindray Biomedical Electronics Co., Ltd.). Serum ADA and ALT levels were measured using a peroxidase assay (test kit from Meikang Biotechnology Co., Ltd) and lactate dehydrogenase assay (Beijing Strong Biotechnologies, Inc.), respectively. Both analyses were performed using a HITACHI 7180 biomedical analyzer. IgA, IgG, and IgM levels were measured using a turbidimetric inhibition immunoassay. Anti-human IgA/IgG/IgM antibody was added to the mixture containing the sample and buffer, and an agglutination reaction was produced, which increased the turbidity of the mixture. Quantification of IgA/IgG/IgM was performed using a Konelab clinical chemistry analyzer to detect turbidity at a wavelength of 340 nm. The reference values for ALT and ADA were as follows: ALT < 40 U/L and ADA < 25 U/L [14].

### Flow cytometry

Lymphocyte subsets, including T lymphocytes (CD3^+^), helper T lymphocytes (CD3^+^CD4^+^), killer T lymphocytes (CD3^+^CD8^+^), NK cells (CD3^−^CD (16^+^56)^+^), B lymphocytes (CD3^−^CD19^+^), and activated B lymphocytes (CD19^+^CD23^+^), were detected using flow cytometry. Peripheral blood samples were labeled with antibodies, including anti-CD3-fluorescein isothiocyanate, anti-CD4-phycoerythrin cyanin 7, anti-CD8-allophycocyanin-cyanin7, anti-CD45-peridin chlorophyll alpha protein-cyanin5.5, anti-CD16^+^CD56^+^ phycoerythrin, anti-CD19-APC, and anti-CD23-Fc EpsilonR II. EDTA-anticoagulated blood (100 µL) mixed with fluorescent-labeled antibodies was incubated in the dark at 18–20 ℃ for 15 min, and 1 mL of red blood cell lysis solution was then added to it. After red blood cells were lysed, centrifuged, and washed twice by centrifugation, the proportion of each lymphocyte subpopulation was detected using a multi-color flow cytometer (BD FACSCanto II) [10]. The results were calculated as a percentage of the positivity rate.

### Indirect immunofluorescence (IIF) assay for EBV-specific antibodies

Venous blood samples (2 mL) were collected and centrifuged after clotting, and the supernatant was collected for subsequent experiments. Specific antibodies against EBV were detected using an IIF assay; anti-VCA IgG/IgM, anti-EBNA IgG, and anti-early antigen IgG IIF kits (EUROIMMUN, Lübeck, Germany) were used for detection. The assay was performed according to the manufacturer’s instructions. EBV-VCA-specific IgG affinity was determined by comparing the fluorescence intensity differences between treatments with and without urea. The difference was < 2 and ≥ 2 grades for high and low affinities, respectively.

### Plasma EBV-DNA PCR assay

EDTA-anticoagulated blood (1–2 mL) was obtained and centrifuged, and the plasma was carefully harvested. A 10 µL aliquot of plasma was mixed with 10 µL of DNA extract (Shengxiang Biotechnology Co., Ltd. Hunan, China) and 40 µL of the PCR mixture, followed by centrifugation. Real-time quantitative PCR was performed using a LightCycler 480II instrument (Roche, Basel, Switzerland). The operation steps were as follows: 50 °C for 2 min and 94 °C for 2 min, followed by 45 cycles of 94 °C for 5 s, 57 °C for 30 s, and 25 °C for 10 s. EBV positivity was defined as a cycle threshold value ≤ 39 (DNA copy number > 400 copies/mL) [14].

All methods were performed in accordance with the manufacturer’s instructions.

### Statistical analyses

Data are presented as numbers (percentages), medians (interquartile ranges), or means ± standard deviations. Analysis of variance and the Kruskal-Wallis test were used for continuous variables. Categorical variables were analyzed using the chi-square test. Spearman’s correlation analysis was used to determine correlations between discrete variables. All statistical analyses were performed using SPSS version 25 (IBM Corp., Armonk, NY, USA) and GraphPad 9.0 Software for drawing. *P*-values < 0.01 were considered statistically significant.

## Results

### Patient characteristics

A total of 104 patients with EBV-associated RTI (EBV-RTI), 32 patients with atypical EBV infection, 54 patients with EBV-IM1 (normal ALT levels), 50 patients with EBV-IM2 (elevated ALT levels), 50 patients with acute respiratory infection (AURI, without EBV infection), and 30 healthy controls were enrolled in this study. There were no statistically significant differences in sex or age between groups (*P* > 0.05). The differences in white blood cell (WBC), lymphocyte counts, ADA levels, IgA, IgG, and IgM titers, percentage of CD3^+^, CD3^+^CD4^+^, CD3^+^CD8^+^, CD16^+^CD56^+^, CD3^−^CD19^+^, and CD19^+^CD23^+^ lymphocytes, and CD4^+^/CD8^+^ ratio between the groups were statistically significant (*P* < 0.01) (Table 1).

**Table 1:** General characteristics of participants

Parameters	EBV-RTI (n = 104)	Atypical EBV infection (n = 32)	EBV-IM1 (n = 54)	EBV-IM2 (n = 50)	AURI (n = 50)	Control (n = 30)	*P*
Male(sex)	55(52.9)	17(53.1)	23(42.6)	24(48.0)	27(54.0)	16(53.3)	0.728
Age(years)	2.9(1.8–4.2)	3.0(1.8–5.1)	3.2(1.9–4.4)	2.8(2.0–4.0)	3.2(2.2–5.1)	3.0(2.3–4.1)	0.812
WBC (⋅10^9^/L)	10.4(7.2–14.1)^a^	11.7(8.0-14.5)^ab^	15.3(11.4–19.5)^bc^	17.2(12.8–23.3)^c^	13.7(9.3–19.3)^bc^	9.1(7.4–12.0)^a^	＜0.01
Lymphocyte count(⋅10^9^/L)	3.7(2.6–6.3)^a^	7.2(4.9–10.5)^b^	10.4(6.4–12.7)^b^	11.8(7.4–16.1)^b^	3.4(2.3–5.2)^a^	3.6(2.8–4.1)^a^	＜0.01
ADA(U/L)	23.7(19.3–34.5)^a^	46.7(39.4–54.5)^b^	45.1(38.9–49.4)^b^	62.3(53.4–79.3)^c^	20.1(17.1–23.8)^ad^	15.9(14.8–18.0)^d^	＜0.01
IgA(g/L)	0.8(0.4–1.3)^a^	1.3(0.9-2.0)^b^	1.3(1.0-1.8)^b^	1.5(1.0–2.0)^b^	0.8(0.5–1.2)^a^	0.7(0.5–1.1)^a^	＜0.01
IgG(g/L)	9.1 ± 3.0^a^	10.6 ± 3.1^b^	10.4 ± 2.5^b^	12.2 ± 2.8^c^	7.7 ± 2.2^d^	7.6 ± 1.4^d^	＜0.01
IgM(g/L)	1.2(0.9–1.6)^ab^	1.2(1.1–1.7)^ab^	1.4(1.1-2.0)^bc^	1.8(1.4–2.2)^c^	1.1(0.8–1.6)^a^	1.1(1.0-1.4)^a^	＜0.01
CD3^+^ (%)	66.3(60.2–74.0)^a^	76.0(70.1–80.3)^bc^	78.1(71.0-82.8)^b^	79.4(75.0-82.7)^b^	63.8(54.1–71.8)^a^	72.5(68.5–73.6)^ac^	＜0.01
CD3^+^CD4^+^ (%)	27.9(21.3–35.0)^a^	19.1(16.9–24.2)^b^	19.1(14.5–23.3)^b^	15.6(12.6–20.6)^b^	32.1(24.8–39.2)^ac^	34.4(33.4–43.4)^c^	＜0.01
CD3^+^CD8^+^ (%)	33.5 ± 13.4^a^	47.8 ± 10.2^b^	51.6 ± 10.1^b^	56.3 ± 11.4^c^	25.0 ± 8.0^d^	25.6 ± 6.1^d^	＜0.01
CD4^+^/CD8^+^	0.9(0.5–1.3)^a^	0.4(0.3–0.5)^b^	0.4(0.3–0.5)^b^	0.3(0.2–0.4)^b^	1.2(1.0-1.7)^c^	1.4(1.25–1.6)^c^	＜0.01
CD3^−^CD(16^+^56)^+^(%)	10.3(7.2–14.9)^ab^	12.9(8.4–17.5)^a^	11.8(7.7–16.9)^ab^	11.9(9.0–17.0)^a^	11.1(6.7–16.1)^ab^	8.8(8.1–9.8)^b^	0.008
CD3^−^CD19^+^ (%)	17.8(11.7–27.0)^a^	10.6(6.1–12.1)^b^	8.1(5.4–11.8)^b^	7.1(4.4–9.4)^b^	20.9(16.0-29.8)^a^	19.8(16.2–21.0)^a^	＜0.01
CD19^+^CD23^+^ (%)	8.3(5.4–12.5)^a^	4.9(2.7–7.1)^b^	4.4(2.4–6.4)^b^	3.5(2.4–6.1)^b^	9.9(6.8–12.7)^a^	9.7(8.0–11.0)a	＜0.01

Values are expressed as n (%), mean ± standard deviation or median (interquartile range). Abbreviation: WBC: white blood cell, ADA: adenosine deaminase; EBV: Epstein–Barr virus; EBV-RTI: EBV associated respiratory tract infection; IM: infectious mononucleosis; AURI: acute upper respiratory infection. *P* < 0.01 between a, b, c and d

### WBC count, lymphocyte count, and ADA level in EBV-related diseases

**Fig. 1:**
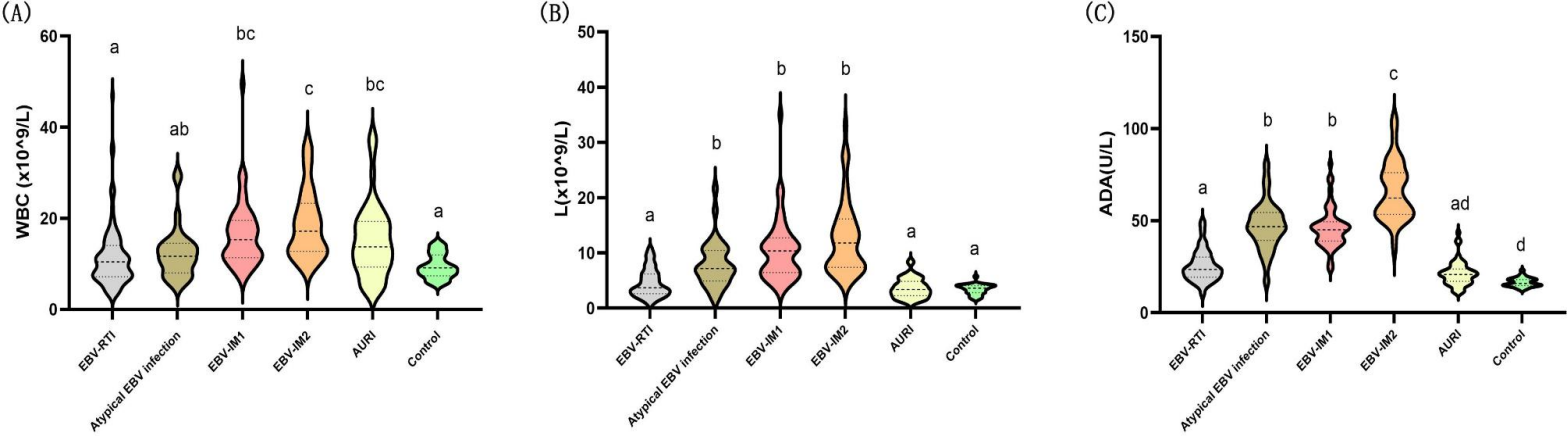
Values of the indicators in EBV-associated diseases. **(A)** WBC count in each group. **(B)** L in each group. **(C)** ADA level in each group. EBV: Epstein-Barr virus, WBC: white blood cell, L: lymphocyte count, ADA: adenosine deaminase, IM: infectious mononucleosis, RTI: respiratory tract infection, AURI: acute infectious diseases. *P* < 0.01 between a, b, c, and d

The WBC count in the EBV-IM2 group was significantly higher than that in the EBV-RTI and atypical EBV infection groups (*P* < 0.01) (Fig. 1). The lymphocyte count and ADA levels in the atypical EBV infection, EBV-IM1, and EBV-IM2 groups were significantly higher than those in the EBV-RTI, AURI, and control groups (*P* < 0.01). Among these, the EBV-IM2 group had the highest level.

### IgA, IgM, and IgG titers in EBV-related diseases

**Fig. 2:**
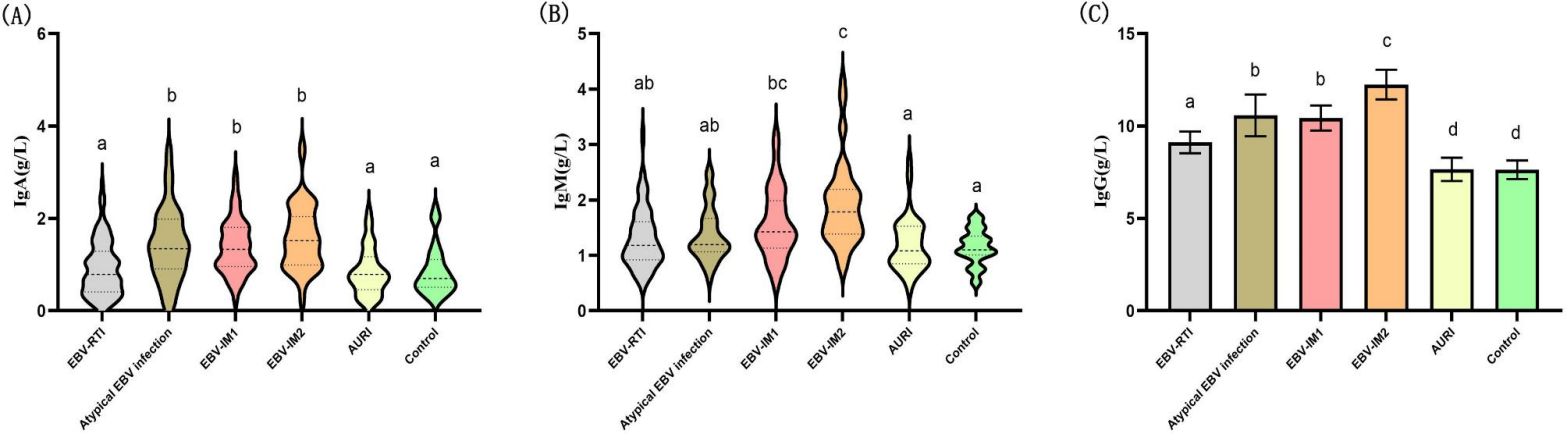
Immunoglobulin titer in EBV-related diseases. **(A)** IgA titer in each group. **(B)** IgM titer in each group. **(C)** IgG titer in each group. ADA: adenosine deaminase, EBV: Epstein-Barr virus, IM: infectious mononucleosis, RTI: respiratory tract infection, AURI: acute infectious diseases. *P* < 0.01 between a, b, c, and d

As shown in Fig. 2, the IgA titer in the atypical EBV infection, EBV-IM1, and EBV-IM2 groups were significantly higher than those in the EBV-RTI, AURI, and control groups (*P* < 0.01). The IgM titer in the EBV-IM2 group was significantly higher than those in the other groups (*P* < 0.01). The IgG titer was the highest in the EBV-IM2 group, and the lowest in the AURI and control groups.

### Lymphocyte subsets in EBV-related diseases

**Fig. 3:**
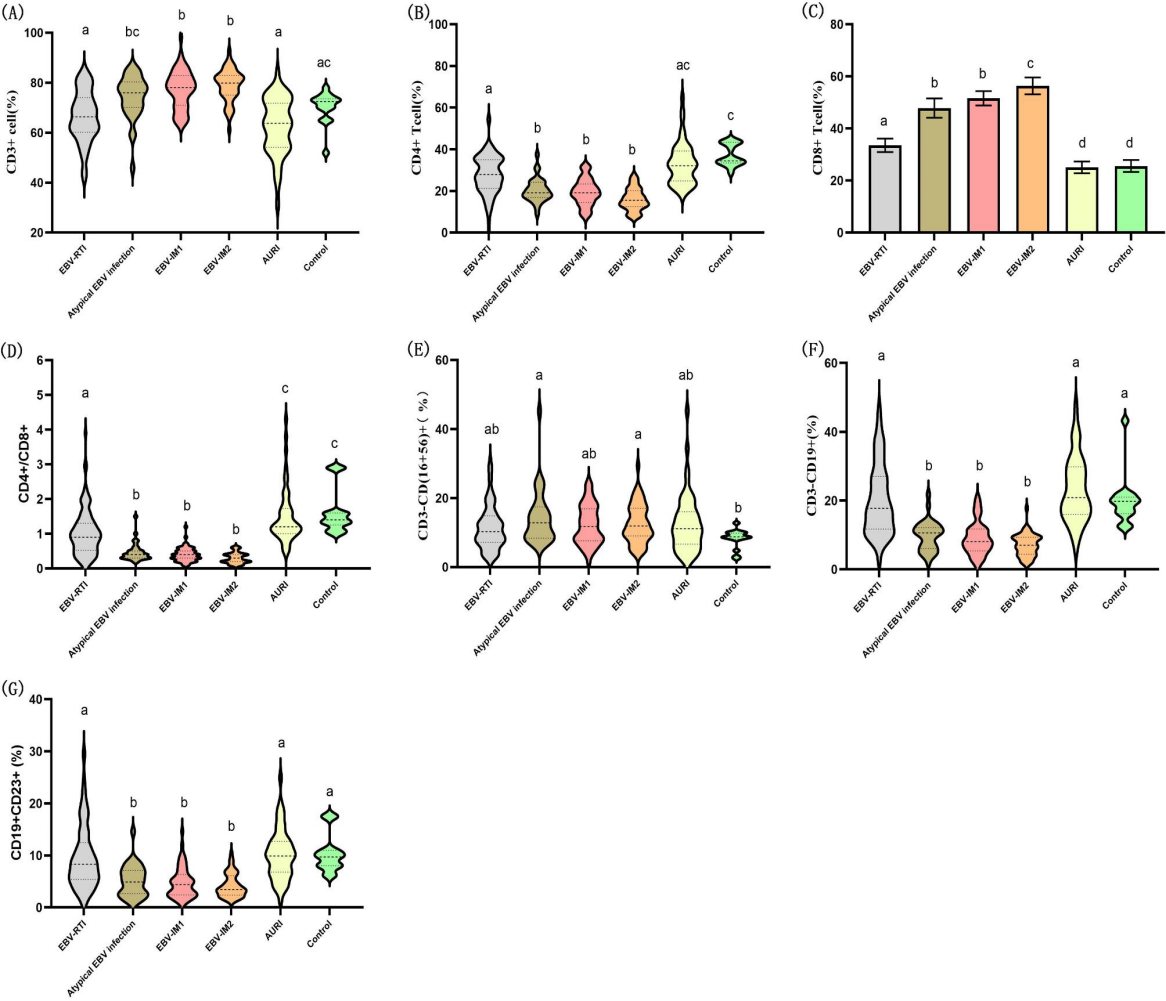
Percentage of lymphocyte subsets in EBV-related diseases. **(A)** Percentage of CD3^+^ cells in each group. **(B)** Percentage of CD4^+^ cells in each group. **(C)** Percentage of CD8^+^ cells in each group. **(D)** CD4^+^/CD8^+^ ratio in each group. **(E)** Percentage of CD3^–^CD(16^+^CD56)^+^ cells in each group. **(F)** Percentage of CD3^–^CD19^+^ cells in each group. **(G)** Percentage of CD19^+^CD23^+^ cells in each group. ADA: adenosine deaminase, EBV: Epstein-Barr virus, IM: infectious mononucleosis, RTI: respiratory tract infection, AURI: acute infectious diseases. *P* < 0.01 between a, b, c, and d

As shown in Fig. 3, the percentages of CD3^+^ and CD3^+^CD8^+^ lymphocytes in the atypical EBV infection, EBV-IM1, and EBV-IM2 groups were significantly higher than those in the EBV-RTI, AUR1, and control groups (*P* < 0.01). However, the percentages of CD3^+^CD4^+^, CD3^−^CD19+, and CD19^+^CD23^+^ lymphocytes and the CD4^+^/CD8^+^ ratio were lower in the atypical EBV infection, EBV-IM1, and EBV-IM2 groups than those in the EBV-RTI, AURI, and control groups (*P* < 0.01).

### Correlation of ADA with Igs and lymphocyte subsets

**Fig. 4:**
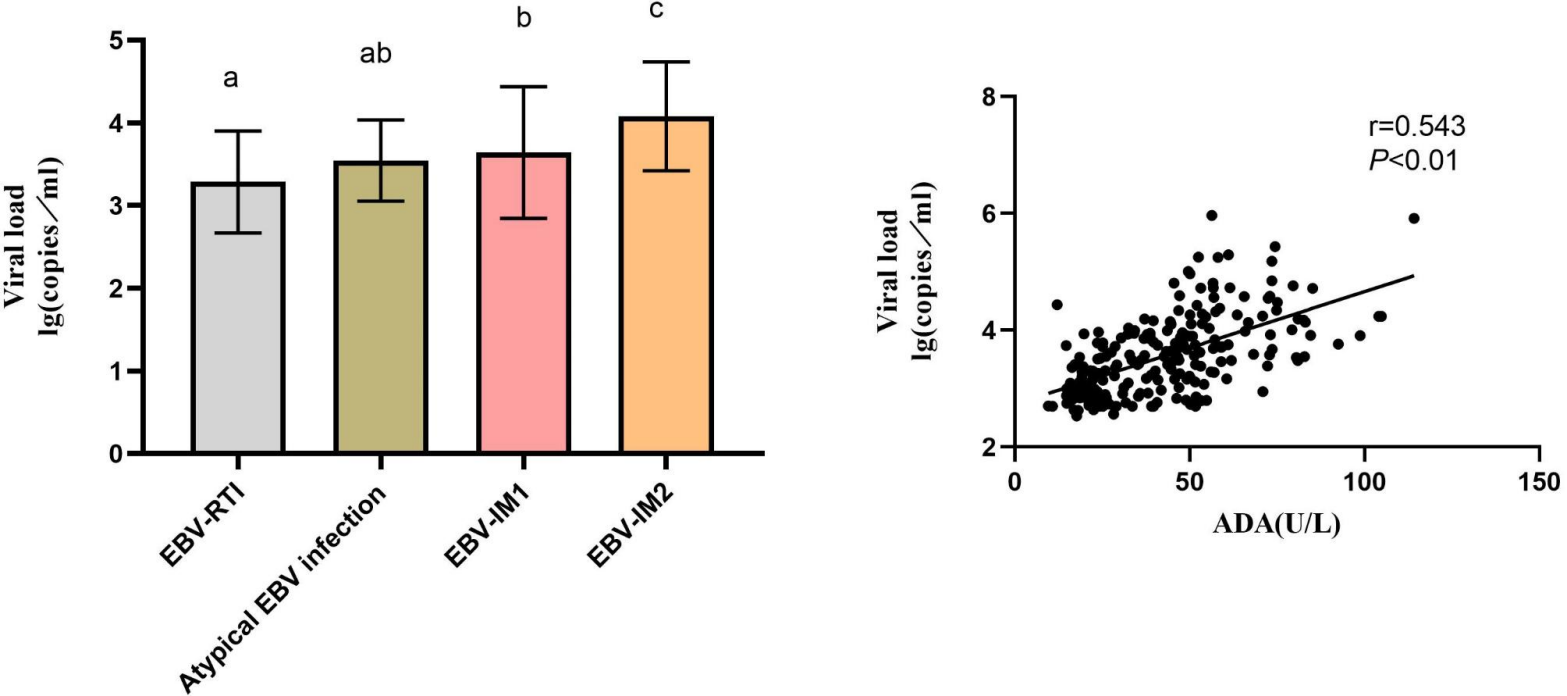
Comparison of viral load in EBV-related diseases **(A)** and correlation analysis of EBV load and ADA **(B)**. EBV: Epstein-Barr virus, ADA: adenosine deaminase, IM: infectious mononucleosis, RTI: respiratory tract infection, AURI: acute infectious diseases. *P* < 0.01 between a, b, and c

In EBV-RTI, the ADA level was correlated with the lymphocyte count; IgA and IgG titer; percentage of CD3^+^, CD3^+^CD4^+^, CD3^+^CD8^+^, CD19^+^CD23^+^, CD3^–^CD19^+^, and CD3^+^CD8^+^ lymphocytes; and CD4^+^/CD8^+^ ratio (r > 0.7, *p* < 0.01) (Table 2). In atypical EBV infections, ADA level was correlated with the IgM titer and percentage of CD3^+^, CD3^+^CD8^+^, and CD3^–^CD19^+^ lymphocytes (*p* < 0.01). In EBV-IM1, ADA levels were correlated with IgG and IgM titers; percentage of CD3^+^, CD3^+^CD4^+^, CD3^+^CD8^+^, CD19^+^CD23^+^, and CD3^–^CD19^+^ lymphocytes; and CD4^+^/CD8^+^ ratio, showing significant correlation with IgM titer and CD3^+^CD8^+^ lymphocyte percentage (r > 0.5, *p* < 0.01). However, ADA level was only correlated with the percentage of CD3^+^CD4^+^, CD3^+^CD8^+^, and CD3^–^CD19^+^ lymphocytes and CD4^+^/CD8^+^ ratio in the EBV-IM2 group (*p* < 0.01). Surprisingly, ADA levels did not correlate with the above indicators in the AURI group. In addition, the IM1 and IM2 groups had higher EBV loads than the EBV-RTI group (Fig. 4). Moreover, ADA level was closely related to the EBV load (r = 0.543, *P* < 0.01).

**Table 2:** Correlation between ADA and other variables in different disease groups

Variable	EBV-RTI			Atypical EBV infection			EBV-IM1			EBV-IM2			AURI	
	r	*P*		r	*P*		r	*P*		r	*P*		r	*P*
L(⋅10^9^/L)	0.330	0.001		0.037	0.840		0.296	0.030		0.427	0.002		-0.012	0.933
IgA(g/L)	0.398	＜0.001		0.340	0.057		0.307	0.024		0.172	0.233		-0.218	0.128
IgG(g/L)	0.391	＜0.001		0.436	0.013		0.521	＜0.001		0.221	0.124		-0.071	0.623
IgM(g/L)	0.250	0.011		0.499	0.004		0.624	＜0.001		-0.118	0.413		0.190	0.187
CD3+ (%)	0.504	＜0.001		0.415	0.018		0.380	0.005		0.246	0.085		0.069	0.635
CD3 + CD4+ (%)	-0.475	＜0.001		-0.085	0.643		-0.361	0.007		-0.369	0.008		0.329	0.02
CD3 + CD8+ (%)	0.718	＜0.001		0.462	0.008		0.536	＜0.001		0.404	0.004		-0.103	0.476
CD4+/CD8+	-0.463	＜0.001		-0.365	0.04		-0.463	＜0.001		-0.481	＜0.001		0.190	0.187
CD3-CD19+ (%)	-0.562	＜0.001		-0.498	0.004		-0.469	＜0.001		-0.450	0.001		-0.137	0.342
CD19 + CD23+ (%)	-0.379	＜0.001		-0.436	0.013		-0.399	0.003		-0.126	0.384		0.026	0.859

Spearman correlation analysis was used. Abbreviation: r: correlation coefficient; L: Lymphocyte count; ADA: adenosine deaminase; EBV: Epstein–Barr virus; EBV-RTI: EBV associated respiratory tract infection; IM: infectious mononucleosis; AURI: acute upper respiratory infection. *P* < 0.01 was statistical significance

## Discussion

Peripheral WBC and lymphocyte counts vary in IM, EBV-RTI, and atypical EBV infections, which are the most common clinical manifestations of EBV infection in children. WBC and lymphocyte counts are not elevated in children with EBV-RTI, whereas they are markedly elevated in IM and are the highest in IM combined with liver impairment. Reportedly, the proportion of cytotoxic CD8^+^ T lymphocytes is elevated in response to activation by EBV surface antigens [16]. Jayasooriya et al. [17] showed that asymptomatic EBV infection in children elicited a virus-specific CD8^+^ T cell without overexpansion; conversely, in IM, CD8^+^ T cell overexpansion was observed. This implies that EBV-RTI may not cause excessive lymphocyte expansion, which is in line with the observation in asymptomatic infected individuals. In addition, this study found no distinction between respiratory infections caused by EBV and other pathogens in terms of lymphocyte counts. This illustrates that acute febrile EBV-induced respiratory illness is not distinguishable from illness caused by other viral pathogens in the routine blood assays [6].

In acute EBV infection, the body successively produces IgM and IgG antibodies against VCA, which can reflect the status of the EBV infection [18]. However, they did not contain protective antibodies. In addition, Sutton et al. [19–22] reported that various autoantibodies were detected in the acute-phase serum of patients with IM as well as the probability of development of autoimmune disorders after IM. In this study, the titers of IgA, IgG, and IgM in children with IM were significantly higher than those in children with EBV-RTI, which further indicated that individuals with IM had a more intense humoral immune response.

T-cell-mediated immune responses play an important role in controlling EBV infection, especially CD8^+^ T cells, which primarily recognize lytic EBV antigens for amplification [7]. CD4^+^ T cells play a crucial role in pathogen elimination by assisting innate and CD8^+^ T- and B-cell-mediated immune responses. Although the CD4^+^ T cell count is not substantially increased during IM, existing data support the concept that CD4^+^ T cells recognize several lytic antigens and, thus, are important contributors to the control of EBV [23]. Therefore, the CD4^+^/CD8^+^ ratio is inverted in IM [24–26]. An elevated CD8^+^ T cell count and inverted CD4^+^/CD8^+^ ratio were observed in all patients with EBV-associated diseases in this study; however, the values varied between different diseases. IM, particularly IM with liver impairment, has a higher CD8^+^ lymphocyte count and more severe CD4^+^/CD8^+^ inversion than EBV-RTI. Recent data implicate NK cells as a prominent factor in the early control of EBV infection through direct cytolysis of infected cells and blockade of transformation via IFN-γ [27]. Furthermore, NK cells derived from tonsillar tissue are more efficient than those isolated from the peripheral blood [28]. NK cells in the peripheral blood did not behave consistently with the other lymphocytes such as CD4^+^ and CD8^+^ T cells in EBV-associated diseases in this study.

ADA is abundant in lymphoid tissues and is considered a marker of immune activation of T lymphocytes. The binding of ADA to CD26 molecules can reduce adenosine concentration and promote the proliferation of T lymphocytes [29]. Moreover, a recent study [30] found that ADA links the adenosine receptor A2AR on the surface of dendritic cells and CD26 on the surface of T cells to form a CD26-ADA-A2AR complex, triggering costimulatory effects that induce the cytokines IL6, IFN-γ, and TNF-α. In this study, ADA level was elevated in EBV-associated diseases but not in AURI with other pathogens, and the elevation was more prominent in IM than in other diseases, particularly when IM was associated with liver impairment. In addition, ADA levels were positively correlated with the EBV load. Therefore, it was speculated that EBV may cause ADA level elevation and that ADA is involved in the immune mechanism of EBV infection. LaMontagne et al. [31] demonstrated that EBV-encoded EBNA1 can bind to the promoter upstream of the ADA gene to promote ADA expression intracellularly, which further confirmed our speculation. In the correlation analysis, ADA was associated with Igs and lymphocyte subsets in EBV-RTI, atypical EBV infection, and IM. However, in patients with IM with transaminase abnormalities, ADA was mainly associated with lymphocytes. This illustrates that ADA acts primarily through cellular immunity in severe EBV-associated diseases.

## Conclusion

First, WBC and lymphocyte counts and ADA, Ig, and lymphocyte subset levels vary in different EBV-associated diseases. Second, IM, especially IM with liver impairment, has higher levels of ADA, Igs, and CD8^+^ T cells and a more pronounced inverted CD4^+^/CD8^+^ ratio than EBV-RTI. Finally, EBV infection can elevate ADA levels. The higher the disease severity caused by EBV, the higher the ADA level, which may be related to its involvement in cellular immune activation.

## Data Availability

The data that support the findings of this study are available from the corresponding author upon reasonable request (2,231,365,607@qq.com).

## List of abbreviations

ADA: adenosine deaminase
ALT: alanine aminotransferase
AURI: acute respiratory infection
EBNA: Epstein–Barr virus nuclear antigen
EBV: Epstein–Barr virus
IgA: immunoglobulin A
IgG: immunoglobulin G
IgM: immunoglobulin M
IIF: indirect immunofluorescence
IM: infectious mononucleosis
NK: natural killer
RTI: respiratory tract infection
VCA: viral capsid antigen
